# Generative Adversarial Network Model to Classify Human Induced Pluripotent Stem Cell-Cardiomyocytes based on Maturation Level

**DOI:** 10.21203/rs.3.rs-4061531/v1

**Published:** 2024-03-11

**Authors:** Ziqian Wu, Jiyoon Park, Paul R. Steiner, Bo Zhu, John X.J. Zhang

**Affiliations:** Thayer School of Engineering, Dartmouth College, Hanover, NH USA; Thayer School of Engineering, Dartmouth College, Hanover, NH USA; Dartmouth Hitchcock Medical Center, Lebanon, NH USA.; Department of Computer Science, Dartmouth College, Hanover, NH USA. He is now with the School of Interactive Computing, Georgia Institute of Technology, GA USA.; Thayer School of Engineering, Dartmouth College, Hanover, NH USA

**Keywords:** Machine Learning, Classication, Cardiomyocyte Maturation, Generative Adversarial Network

## Abstract

**Objective:**

Our study develops a generative adversarial network (GAN)-based method that generates faithful synthetic image data of human cardiomyocytes at varying stages in their maturation process, as a tool to significantly enhance the classification accuracy of cells and ultimately assist the throughput of computational analysis of cellular structure and functions.

**Methods:**

Human induced pluripotent stem cell derived cardiomyocytes (hiPSC-CMs) were cultured on micropatterned collagen coated hydrogels of physiological stiffnesses to facilitate maturation and optical measurements were performed for their structural and functional analyses. Control groups were cultured on collagen coated glass well plates. These image recordings were used as the real data to train the GAN model.

**Results:**

The results show the GAN approach is able to replicate true features from the real data, and inclusion of such synthetic data significantly improves the classification accuracy compared to usage of only real experimental data that is often limited in scale and diversity.

**Conclusion:**

The proposed model outperformed four conventional machine learning algorithms with respect to improved data generalization ability and data classification accuracy by incorporating synthetic data.

**Significance:**

This work demonstrates the importance of integrating synthetic data in situations where there are limited sample sizes and thus, effectively addresses the challenges imposed by data availability.

## I. INTRODUCTION

Human induced pluripotent stem cell-derived cardiomyocytes (hiPSC-CMs) have emerged as a promising tool for drug testing, disease modeling, and tissue replacement for cardiovascular medicine due to their unlimited and personalized, patient-specific source. HiPSC-CMs are now generated in vitro from personalized cell sources with high throughput and purity at a clinically relevant scale, but a major hurdle halting their advancement to clinical research phase comes from their immature, embryonic state. Embryonic cardiomyocytes undergo significant developmental changes during postnatal stages, including subcellular structural development, improved calcium handling and changes in action potential profile [1]–[6]. The current protocols generate hiPSC-CMs at embryonic or early fetal stages and thus, the generated cells lack many attributes of adult cells that are desirable for drug screening, modeling of adult-onset diseases, or replacing cells lost to disease. Therefore, many studies have been invested in developing methodologies and tools to accelerate maturation of hiPSC-CMs via biophysical or chemical stimuli such as mechanical loading [7]–[9] or electrical stimulation [10]–[12], optical stimulation [13], [14], biochemical and biophysical cues [15]–[17], but the throughput and scalability of current experimental designs and analysis methods are still limiting in scope to manufacture mature hiPSC-CMs at scale and at speed. The standard state-of-art approaches to evaluate the structural or functional state of cardiomyocytes are mostly based on video recording and microscopy image analysis [18]–[20], but the key unresolved challenge is acquiring large sets of image data and high throughput means to process, analyze, and classify image features.

The ability to manufacture mature hiPSC-CMs at scale and at speed will utterly transform traditional health care to one with greater focus on regenerative medicine and cell therapies. It also has the potential to shift the pharmaceutical industry as the use of novel human cell-based assays supports the industry-wide mandate to reduce, refine, and replace animal testing. Core innovations to achieve this will come from scalable and reproducible maturation of hiPSC-CMs through low-cost and high throughput means and development of a standardized and robust framework for conducting cellular measurements and analyses. Analysis of cellular systems have faced many challenges due to high degrees of complex variabilities and lack of sufficient experimental data, but integration of artificial intelligence (AI) and computational modeling has forged a paradigm shift towards high throughput analysis and physical principle based, data-driven accurate predictions. Towards this effort, we herein introduce a generative artificial intelligence method that generates synthetic hiPSC-CM image data that closely resembles the real hiPSC-CM image data in order to enlarge the dataset used for high throughput, comprehensive analysis on classifying maturation features with respect to specific experimental conditions.

Machine learning models are renowned for their exceptional performance when handling intricate and high-dimensional data with diverse attributes. These models possess the ability to uncover the inherent features present in large datasets even in the absence of an understanding of the underlying mechanism governing the system. This ability proves particularly valuable for analyzing complex systems that lack mathematical descriptions of their dynamics, such as the maturation process of fetal to adult cardiomyocytes. The broad application of machine learning models encompasses various cell classification tasks – for instance, the classification of cancer cells [21]–[27]. Convolutional neural network (CNN) is an algorithm type that is useful for such cell classification applications due to its ability to extract intrinsic features and patterns from images and therefore, eliminates the need for laborious and manual image analysis. However, a large and diverse training dataset is typically required for machine learning models to ensure accurate and reliable classification and be able to grasp the full complexity of the cellular system dynamics. Data limitation in size and scope can create biases in machine learning models and lead to overfitted outcomes. Training on a small or biased dataset can render a model with limited ability to make autonomous predictions with a new and varied data set.

Generative AI offers a viable solution for limited experimental datasets by creating synthetic data that reproduces the characteristics of real data. It was not until 2014 that the introduction of generative adversarial networks (GANs) enabled producing high-quality data of human facial features that are convincingly authentic [28]. The GAN algorithm employs two neural networks - a generator and a discriminator - to generate synthetic data that closely resembles the original data. The generator creates artificial data while the discriminator attempts to differentiate between the synthetic data and the real data. The two networks engage in an adversarial training process: the generator is trained to produce synthetic data that progressively approximates the original data, and the discriminator learns to distinguish fine features between the two. This adversarial training process is capable of enhancing the model’s resilience to adversarial attacks and perturbations in a different dataset. In recent years, GAN models have shown applications in cancer cell classifications [29]–[32] and neural cell classifications[33]. However, to the best of our knowledge, GAN has not yet been utilized in the context of cardiac cellular systems.

Here, we present the development of a GAN-based approach for generating high-fidelity synthetic data that replicate hiPSC-CMs cultured in different microenvironments. This includes synthetic hiPSC-CM images and videos that accurately capture the dynamic behavior of cardiomyocytes. To train the GAN model, a micropatterned hydrogel platform was designed to culture hiPSC-CMs and provide biophysical stimuli to facilitate their maturation, and the control group was cultured in traditional glass well plates. Cells were cultured for 14 days, and optical recordings were collected every other day to analyze their structural and functional behavior over time. Recordings and images collected from day 2, 6, 14 without further image processing or augmentation were used for the GAN application here.

**Figure 1.**
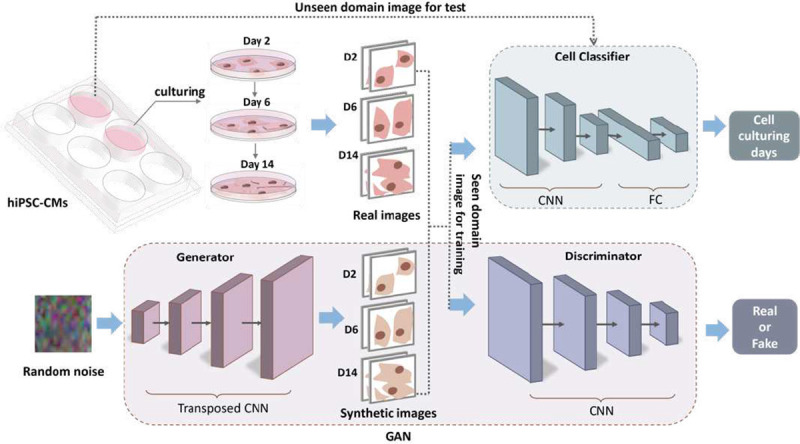
Overview of the GAN model to improve classification of hiPSC-CM maturation level. Morphology images and contractility recordings (seen domain) are collected to train the GAN model to generate high quality artificial data composed of cell images and contractile recordings. Relevant synthetic images trained from the seen domain then are mixed with the real data to train the cell classifier to improve its classification accuracy in both the seen and the unseen domains. Fake videos are obtained by training the GAN model with time series images of individual hiPSC-CMs.

We combined the synthetic cell images produced by the GAN model with the experimental dataset to train a CNN model that can classify hiPSC-CMs at various maturation stages. Cross-validation is a widely used method to evaluate the versatility of machine learning models. However, when dealing with limited datasets, such as in the case of hiPSC-CMs systems, performing cross-validation alone on a limited dataset that lacks population and diversity does not provide sufficient evidence for generalization on the entire data domain. To address this limitation, we prepared an unseen domain dataset (Fig. 1) that consists of data from a different cell batch but under the same culture conditions and thus, shares similar characteristics with the training data but not fully represented by it.

## II. METHODS

### A. Generative Adversarial Network (GAN) Model development to generate synthetic image data

The GAN model employed in this study was comprised of a generator and a discriminator as shown in Fig. 1. The generator applied upsampling techniques to a random noise vector input, and generated synthetic images that closely resembled the original, real image. In contrast, the discriminator functioned as a binomial classifier, downscaling input cell images to discern between real and fake samples. The core of the GAN model lay in its adversarial training approach, wherein the generator and discriminator alternated undergoing iterative updates and compete with each other. The discriminator was trained to minimize a binary classification loss function, while the generator was trained to maximize the probability of the discriminator misclassifying the generated samples. The objective function is described in the following equation:

$$\underset{G_{\theta }}{\min}\;\underset{D_{\phi }}{\max}\;L_{\theta };\phi =\sum \left(\log D_{\phi }(x)+\log\left(1-D_{\phi }\left(G_{\theta }(z)\right)\right)\right)$$

In order to facilitate balanced competition between the generator and discriminator, and otherwise promote impartial learning during adversarial training, the networks were designed with a symmetric structure. The generator consisted of four layers of transposed 2D CNNs, while the discriminator consisted of four layers of 2D CNNs. Both networks incorporated batch normalization and rectified linear unit (ReLU)/Leaky ReLU activations between each layer. The generator concluded with a Tanh activation function, while the discriminator utilized a Sigmoid function. Details of the generator and discriminator structure can be found in Table I. The adversarial training optimized both neural networks, enhancing the model’s robustness for generalization and defense of subtle perturbations in the data.

The GAN model was trained with the objective of producing high-quality artificial hiPSC-CMs data, which included both synthetic images and videos. These generated cell images were combined with authentic data to form the training dataset for the cell classifier model. Inclusion of synthetic cell images served the purpose of improving the scale and diversity of the dataset, which in turn enhanced the accuracy of computational analysis for classifying hiPSC-CM images into various stages of maturation.

### B. Cell Classification Framework

**Figure 2.**
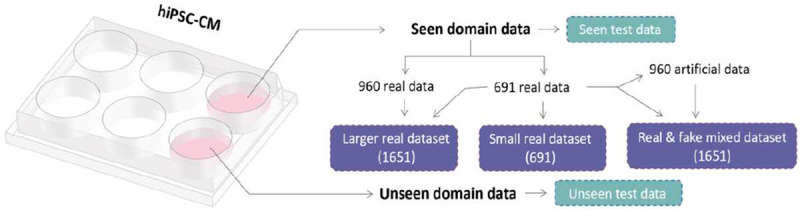
Schematic to show the relationship of the training data and the testing data for the cell classifier.

The cell classifier architecture was constructed with a layered structure that consists of five layers – including three CNN layers and two fully connected (FC) layers (Fig. 2). To investigate the impact of integrating synthetic data into the training dataset, three distinct datasets were curated for the training of the classifier. These datasets included a relatively small authentic dataset, a larger authentic dataset, and a dataset that combined both authentic and synthetic images. The cell classifier underwent testing with both seen and unseen data to evaluate the GAN model’s ability to generate synthetic images that contain detailed features of the cardiac cells that were not sufficiently represented in the experimental dataset due to limited sampling. Since the GAN model was only trained with the seen domain data, this evaluation was intended to demonstrate the GAN model’s ability to generate artificial images that contained features beyond what was present in the original dataset.

To validate the effectiveness of the proposed model, the classification outcomes were compared against four conventional machine learning algorithms: Support Vector Machine (SVM), Random Forest, K Nearest Neighbors (KNN), and Naive Bayes. In order to assess the generalization ability they each achieved from incorporation of synthetic data, each conventional machine learning model was trained using both real and synthetic datasets. Subsequently, those models’ ability to generate synthetic images with novel features were evaluated using both seen and unseen domain testing data.

### C. Fabrication of Micropatterned Hydrogel Scaffolds to Facilitate Maturation of hiPSC-CMs

**Figure 3.**
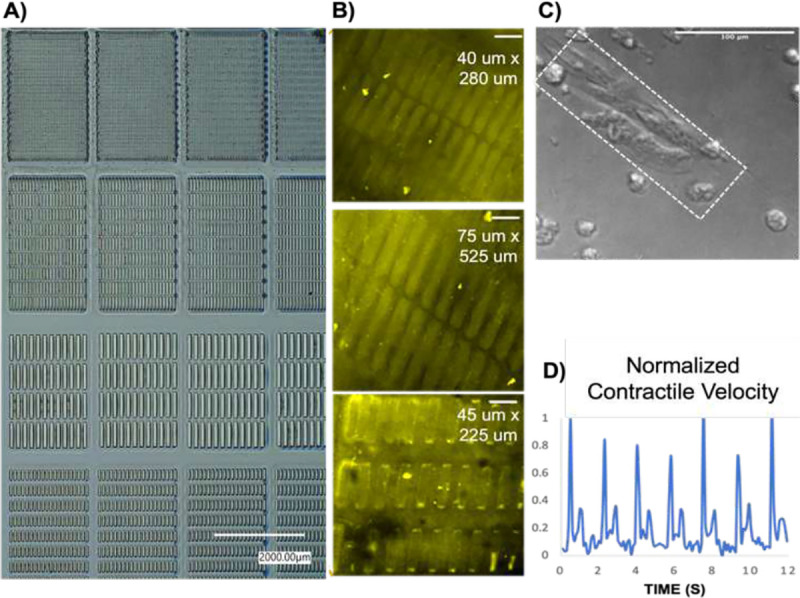
(A) Various micropatterns generated via lithography. (B) Immunostaining of collagen IV coated patterns (scale bars: 100 um) (C) HiPSC-CMs cultured on collagen IV micropatterned GelMA hydrogel scaffold that demonstrate mature morphology (scale bar: 100 um) (D) Motion vector analysis of contractility

To generate maturation-enhanced hiPSC-CMs, they were cultured on a micropatterned, collagen IV coated photosensitive hydrogel with controlled mechanical properties. A 10% (w/v) gelatin methacrylate (GelMA) was combined with 0.5% Irgacure 2959 photoinitiator to generate photo-crosslinked hydrogels. Sterility was ensured via sterile 0.2 μm porous rapid filtration. These hydrogels were casted in a custom Teflon mold and sealed with glass to polymerize under 365nm 8mW/cm2 UV light and subject to varying crosslinking times to generate a stiffness gradient of 10 kPa, 30 kPa, and 60 kPa. Polydimethylsiloxane (PDMS) stamps with micropatterns including 20 um × 140 um 40 um × 280um, 75 um × 525 um, and 45 um × 225 um size rectangular patterns were fabricated using traditional photolithography and soft lithography (Fig. 3A). Plasma-activated PDMS stamps were coated with collagen IV protein and stamped onto the 10% GelMA hydrogel scaffolds (Fig. 3B).For the positive control groups, hiPSC-CMs were cultured on collagen IV coated MatTek glass well plates.

### D. Optical Measurements of Cardiomyocyte Structure and Function

Commercially available human iPSC-derived cardiomyocytes (iCell^2^ cardiomyocytes, 01434) were obtained from Cellular Dynamics International Inc. (CDI, Madison, WI, USA). Cryopreserved iCell^2^ cardiomyocytes were rapidly thawed, then diluted in iCell^2^ plating medium and seeded onto standard 6-well and 96-well plates (Thermo Fisher Scientific) coated with 0.1% gelatin (Sigma Aldrich) for the control groups and on 10% GelMA hydrogel scaffolds coated with collagen type IV proteins for the maturation enhancement group (Fig/ 3C). After 4 hours post seeding, the plating medium was changed to a maintenance medium and then changed every 48 hours thereafter. Cell cultures were maintained in the incubator at 37°C and 5% CO2. The hiPSC-CMs were cultured for two weeks and characterized every other day using a Nikon TE2000 inverted microscope to record the cellular morphology and beating dynamics at 10 frames per second. To assess contractile motion of hiPSC-CMs, movement was quantified using a custom MATLAB script, which measured pixel displacements of contracting cells over contraction and relaxation. For each video frame, the mean magnitude of displacement was measured to yield an average contractile movement. Normalized contractile motion was calculated for each video as the mean of all peak contraction values observed in a 20 second period (Fig. 3D).

### E. Generation of seen domain and unseen domain data

Videos of day 2, day 6 and day 14 hiPSC-derived cardiomyocytes were collected to represent the different stages of the cardiomyocyte maturation process. Images were extracted and randomly cropped from these videos to obtain 300 × 300 pixel of RGB cell images. The collected real images were separated into two groups: cells cultured in one maturation-promoting scaffold included in the seen domain, and cells cultured in another scaffold included in the unseen domain. Both groups of cells were cultured under the same conditions, and the same separation process also was done for the control group. The seen domain dataset was utilized for the training of the GAN and cell classifier, as well as for testing the accuracy of the cell classifier. The unseen domain data was employed for testing the generalization ability of the cell classifier.

### F. Implementation and Training of GAN model

All of our GAN and cell classification models were implemented through Pytorch on a standard workstation (Intel(R) Core(R) CPU i9–9980 XE CPU 3.00 GHz, 18 CPU cores, 8GB NVIDIA GeForce TRX 2080Ti). The Adam optimizer was employed to minimize the loss of the GAN model and a standard error back-propagation algorithm was used, with $\beta_{1}=0.5$ and $\beta_{2}=0.999$. A batch size of 64 was used, and the learning rate was set to 0.0002. The cell classifier underwent training for 2000 epochs. The weights were controlled with weight norm regularization to avoid overfitting.

To generate synthetic images, a dataset of 691 seen domain images was utilized. This dataset consisted of 229 images from day 2, 227 images from day 6, and 235 images from day 14. These images underwent transformations such as random cropping, random flipping, and resizing, resulting in images with a resolution of 128 × 128 pixels and RGB channels. The generator component of the GAN model took a noise vector of size (64,1) as input and generated an image of size (3,128,128) as output. The discriminator, on the other hand, took an image of size (3,128,128) as input and output a probability indicating whether the input image was genuine or artificial. The GAN model was trained for a total of 2000 epochs with the objective of generating 320 images for each class of cardiomyocytes.

To generate synthetic videos that replicate the beating dynamics of cardiomyocytes, a dataset comprising 124 groups of single cell time-series images was collected. Each group consisted of five consecutive frames, with each frame being an RGB image of size 256 × 256 captured at a frame rate of 5 frames per second (FPS). These collected images underwent several transformations, including random cropping, random flipping, grayscale conversion, and resizing, resulting in each group containing five consecutive single-channel cell images of size 64 × 64. The generator component of the GAN model took a noise vector of size (64,1) as input and generated an output vector of size (5,64,64). On the other hand, the discriminator took a vector of size (5,64,64) as input and output a probability indicating whether the input vector represented a genuine beating cell or an artificial beating cell. The GAN model was trained for 2000 epochs using this setup. The generated vector of size (5,64,64) was further transformed into a short synthetic video that replicated the beating of a single cardiomyocyte.

### G. Implementation and Training of the Cell Classifier

The cell classifier architecture was structured with three convolutional neural network (CNN) layers followed by two fully connected (FC) layers. Each CNN layer had a kernel size of 3 and produced output channels of 32, 16, and 4, respectively. Following each CNN layer was a 2D maximum pooling layer with a size of (4,4). The two FC layers had sizes of 64 and 3 respectively, and the classifier concluded with a SoftMax activation layer. The input to the classifier was cardiac cell images, either real or artificial, with dimensions of (3,96,96) that randomly were transformed from the image training dataset with each image of size (3,128,128). The output of the classifier is a vector of length 3, which indicated the probabilities of each class for the input image.

To examine the impact of synthetic images generated by the GAN model, three distinct training datasets were prepared. The first dataset was the original training dataset used for training the GAN model, consisting of 691 real cell images from days 2, 6, and 14. The second dataset combined the images from the first dataset with an additional 960 real images (320 per cell class), resulting in a total of 1651 real cell images. The third dataset combined the 691 real images with 960 synthetic images (320 per cell class), resulting in a total of 1651 mixed real and fake cell images. The relationship among these three training datasets is depicted in Fig. 2. During training of the cell classifier, the Adam optimizer with $\beta_{1}=0.9$ and $\beta_{2}=0.999$ was utilized with a batch size of 64. The learning rate was set to 0.0005. The cell classifier underwent training for 1000 epochs.

## III. RESULTS

### A. Generation of synthetic images of hiPSC-CMs

**Figure 4.**
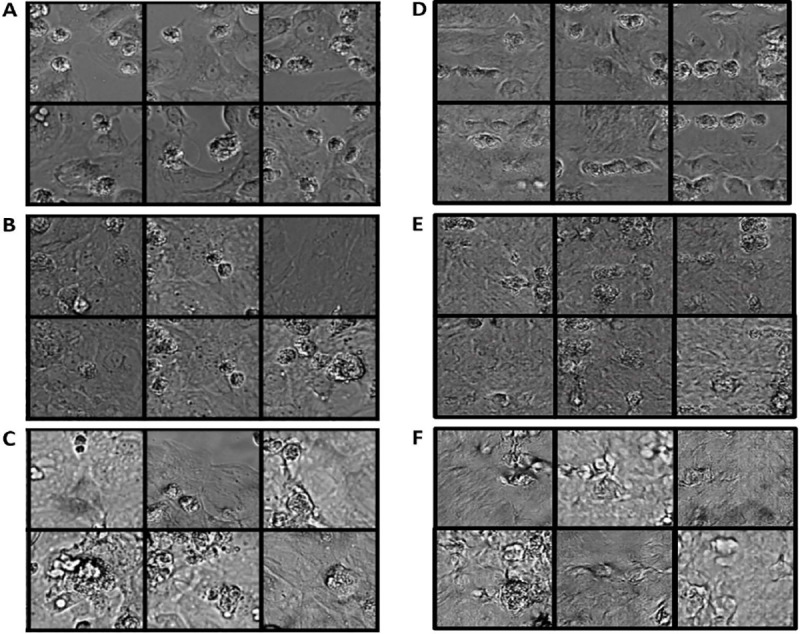
Comparison of real control group hiPSC-CM images and the generated hiPSC-CM images. (A-C) Real images of control group hiPSC-CMs cultured for 2 days (A), 6 days (B), 14 days (C). Synthetic images of hiPSC-CMs that correspond to a 2-day culture (D), 6-day culture (E) and 14-day culture (F).

Fig. 4 presents a visual comparison among the generated images of hiPSC-CMs on day 2, day 6, and day 14, and the corresponding real images of hiPSC-CMs. The results demonstrate that our GAN model is capable of reproducing distinct characteristics observed in human cardiac cells at different stages of culture on a maturation promoting scaffold. Specifically, the synthetic images generated by our GAN model successfully capture the varying patterns exhibited by human cardiac cells at different culture timepoints. For instance, the day 2 synthetic images exhibit a sparse distribution of cells, small cell surface areas, and distinct boundaries between cells. On the other hand, both the real and synthetic images at day 14 exhibit a dense distribution of cells, elongated cellular shapes, and larger cell surface areas. These observations demonstrate the ability of our GAN model to generate synthetic images that accurately replicate the diverse characteristics of human cardiomyocytes during their maturation process.

### B. Generation of synthetic videos of hiPSC-CMs

**Figure 5.**
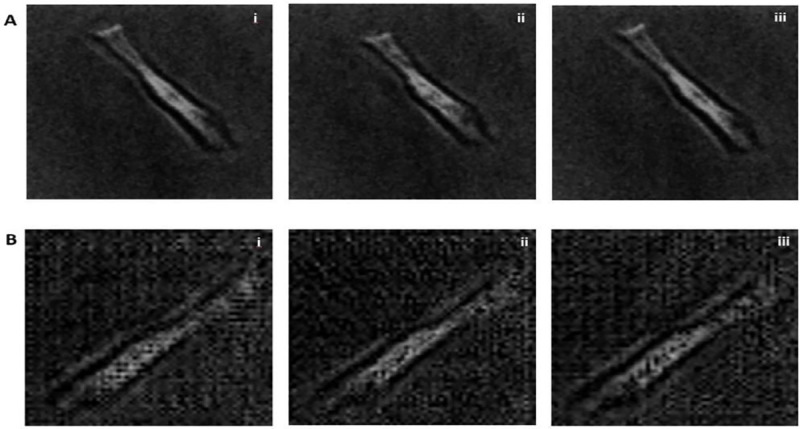
Authentic and synthetic videos of a beating maturity-enhanced single hiPSC-CM. (A) The real time series image of a single beating hiPSC-CM, shown in 256 × 256 pixel resolution. (B) The synthetic time series image of a single beating virtual hiPSC-CM, shown in 64 × 64 pixel resolution. Each image is taken at an interval of 0.5 s from time series (i) to (iii) in both A and B.

Fig. 5 presents a motion video example of a synthetic human cardiomyocyte with a resolution of 64×64 in greyscale. This synthetic video is compared to a real video that depicts the contractile motion of a single aligned hiPSC-CM shown in Fig. 5B. The displayed figure showcases the progression of a single artificially generated cardiomyocyte from a relaxed state (Figure 5Bi) to a contraction state (Figure 5Bii), and subsequently returning to the relaxed state (Figure 5Biii). Notably, these synthetic frames closely mimic the beating dynamics observed in the real hiPSC-CM video shown in Fig. 5A. This visual comparison highlights the capability of our model to generate synthetic cardiomyocyte videos that faithfully reflects the spatiotemporal dynamics exhibited by real cardiomyocytes.

### C. Principal Component Analysis of the generated cell images

Principal Component Analysis (PCA) is a widely adopted methodology used to analyze massive datasets that encompass a substantial number of dimensions or features per observation. This method eases data interpretability while retains critical information, thereby enables effective visualization of multidimensional data. This is accomplished by applying linear and orthogonal transformation of data into a new coordinate system where variation in the data can be described with fewer dimensions compared to the original data. In numerous studies, the first and second principal components have been frequently applied to construct a two-dimensional representation of the data and thus, enabled effective visual identification of clusters that consist of closely related data points[34].

**Figure 6.**
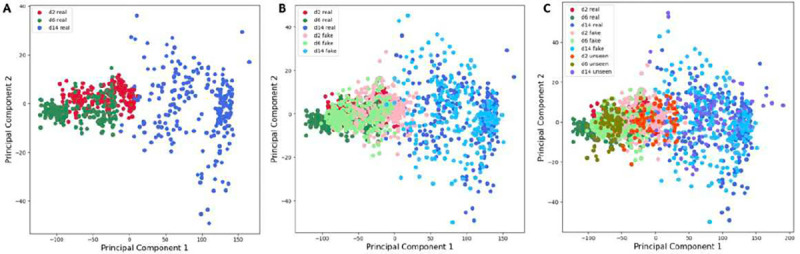
Principal component analysis of the real and fake data

**Figure 7.**
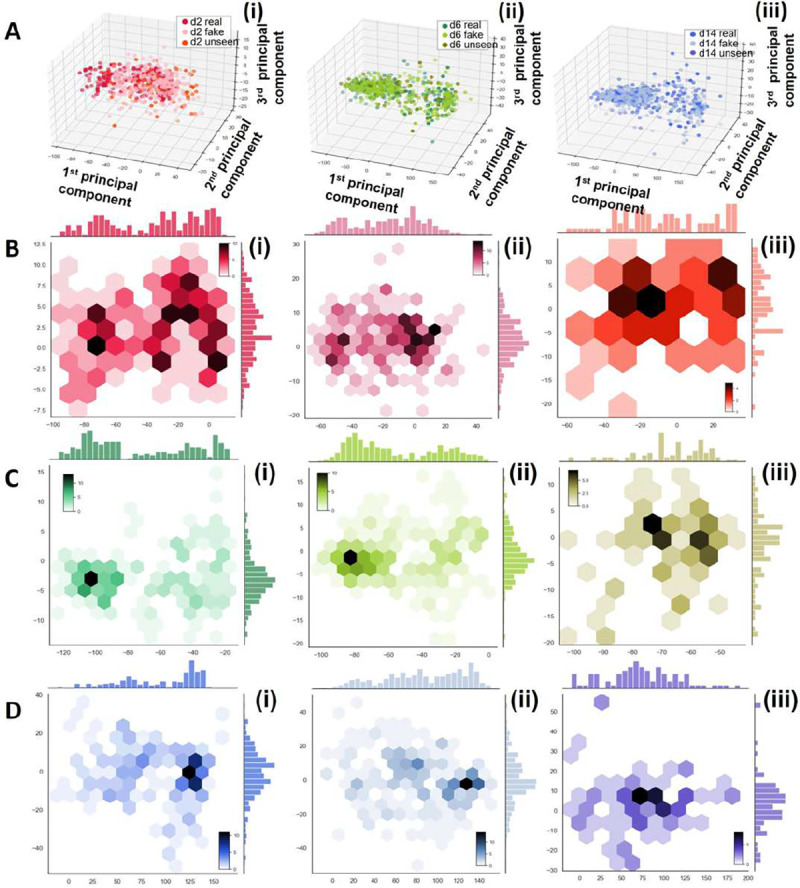
Principal component analysis (PCA) of the real data, fake data, and the unseen data of different cell classes. (A) The first three PCA of (i) day 2 (ii) day 6 and (iii) day 14 hiPSC-CMs. (B)-(D) Hex plot with marginal distributions to show the first two principal components of (B) day 2, (C) day 6, (D) day 14 of the (i) small real data set, (ii) synthetic data, (iii) unseen data.

To quantitatively assess the ability of our proposed GAN model to generate synthetic cell images that exhibit similar features to the authentic ones, we conducted principal component analysis (PCA) to examine the distribution of the underlying main components in both real and synthetic image data. The results are illustrated in Fig. 6 and Fig. 7. Fig. 6A represents the first two principal components obtained from the experimental dataset of 691 samples that were also used to train the GAN model. The analysis reveals that while the images from day 14 exhibit a distinct distribution of features, there is an overlap between the day 2 and day 6 data, indicating that these two classes share similar yet discernible features. Fig. 6B showcases the PCA results of each real and synthetic data, including the real data samples shown in Fig. 6A and the synthetic dataset of 960 samples generated by the GAN model. The findings demonstrate that for each class of cell images, the synthetic data samples display overlapping distributions with the real data, which demonstrate the ability of our GAN model to generate synthetic images that capture real features. Fig. 6C displays the PCA results of the seen real data, fake data, and the unseen real data. While most of the unseen data features are covered by the seen real data, some of the unseen data exhibit outlier features, which can pose challenges for classification models that solely rely on real data with limited sampling. However, this scenario is often encountered in in vitro cardiac systems.

A comprehensive analysis of the principal component analysis (PCA) results for various classes and types of data is presented in Fig. 7. The comparison provides detailed insights on the distribution of features. In Fig. 7B, C, and D, the features of the unseen data are more densely distributed in synthetic images compared to the authentic images. This observation suggests that the GAN model successfully captures the most significant and prominent features from the original data. Additionally, Fig. 7B and 7C reveal that the synthetic data exhibits an expanded feature distribution in the first principal components compared to the authentic data. This expansion allows the synthetic data to cover a wider range of features, including those that may not be prominent in the original data due to limitations in sample size and diversity.

These findings demonstrate the ability of our proposed GAN model to successfully generate synthetic cell images that exhibit similar features to the real ones and thus, improves the data analysis cost and accuracy by aiding in sampling power. The GAN model demonstrates its ability to extract important features from the real data, while also introducing novel features that may not be fully reflected in but based on the original data. This ability to generate synthetic data with greater diversity and broader feature coverage greatly enhances the effectiveness and practicality of our GAN model. This PCA results underscore the challenges and biases of working with limited real data when constructing a classifier with strong generalization potential, especially as the unseen data may contain outlier features. Thus, integrating synthetic data becomes crucial in situations where the availability of experimental data is limiting.

### D. Visualization of the Cell Classifier Feature Maps

To validate the function of our proposed cell classifier, we present the feature maps generated by the trained classifier, which provide a visual representation of the learned CNN features within the classifier.

**Figure 8.**
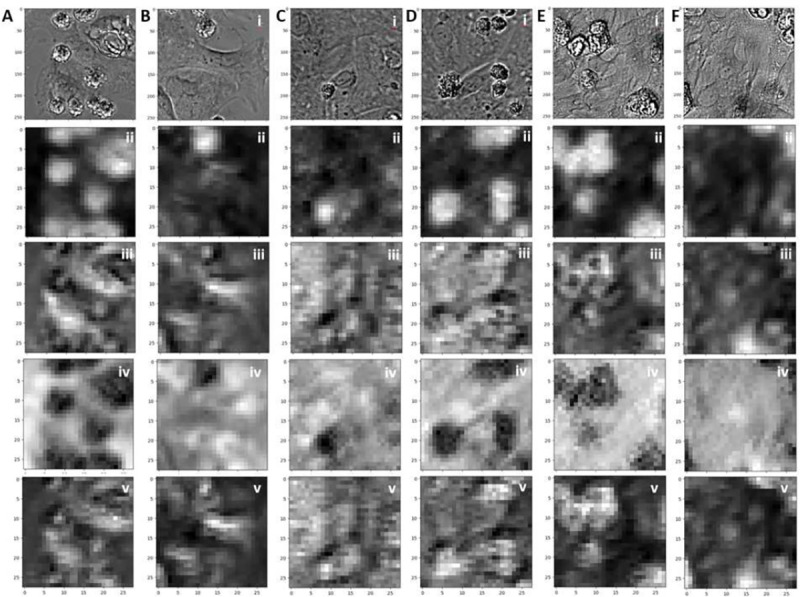
Feature maps of the cell classifier. (A)(B): (i) The input data and the (ii-v) four channel CNN filter feature maps of day 2 control cell images. (C) (D): (i) The input data and the (ii-v) four channel CNN filter feature maps of day 6 control cell images. (E)(F): (i) The input data and the (ii-v) four channel CNN filter feature maps of day 14 control cell images.

Fig. 8 represents both the input images (i) and the resulting four-channel output from the last CNN layer, and each filter mask result is displayed in (ii-v). Specifically, Fig. 8A and 8B correspond to day 2 cell images, Fig. 8C and 8D represent day 6 cell images, and Fig. 8E and 8F depict day 14 cell images from the control group. In the feature map figures, regions that appear brighter indicate a higher activation or presence of the learned feature. While the feature map represents an intermediate outcome of the entire classifier, it offers insights into the internal mechanisms of the CNN within the classifier. For instance, the results from the first filter mask in Fig. 8A(ii) exhibit a strong correlation with the most prominent regions in the cell images, many of which correspond to dead cells. Conversely, the results from the second filter mask in Fig. 8F(ii) demonstrate a strong correlation with the alignment of each cell. These two features play critical roles in distinguishing different classes of hiPSC-CMs solely based on their graphical characteristics. These findings elucidate how our proposed CNN classifier can capture the intricate features of hiPSC-CMs and facilitate accurate classification of cells into various culture stages.

## IV. DISCUSSION

### A. Ablation Test

To validate the accuracy of our CNN cell classifier, we conducted a comparison with models that share similar structures. These include a 5-layer fully connected model, a reduced-size CNN with fewer layers, and a significantly larger CNN model that follows the same structure as our discriminator, which is commonly employed in other studies [30]. The results are presented in Table I. All models were trained using a mixed dataset of real and synthetic samples and were tested on both seen and unseen domain samples.

It is worth mentioning that our proposed model demonstrates nearly the highest accuracy across all testing scenarios, except for the discriminator-structure model, which exhibits higher accuracy in the seen domain tests. However, the difference is insignificant since both methods achieve extremely high accuracy in seen domain tests. Nevertheless, our proposed model significantly outperforms the others in the unseen domain tests. Consequently, we didn’t select the discriminator structure for the cell classification task here.

The disparity between our proposed model’s performance and the others’ performance in the unseen domain tests can be attributed to two reasons. Firstly, our proposed model does not consider the entire image as input. Instead, it selects a random crop of size 96 × 96 from the original image during each training epoch. This approach ensures that our model learns the features of neighboring pixels from fragmented images, thereby enhancing its performance when encountering new data. This is particularly crucial in the case of intact hiPSC-CM images in which cells are closely packed, lack orderly alignment, and vary in cell features from one image to another. This is in contrast to other cell classification tasks that involve distinguishing single cell types, where each cell is positioned at the center of the image to maintain fixed feature positions. This also accounts for our selection of random crops during each training epoch of the GAN model. Consequently, utilizing a discriminator structure that takes the entire image as input would compromise the model’s ability to handle perturbations in novel data. Second, the presence of substantial number of parameters in the discriminator can increase the risk of overfitting the CNN model when trained on a small dataset.

### B. Classification of hiPSC-CMs with different methods and data sets

**Figure 9.**
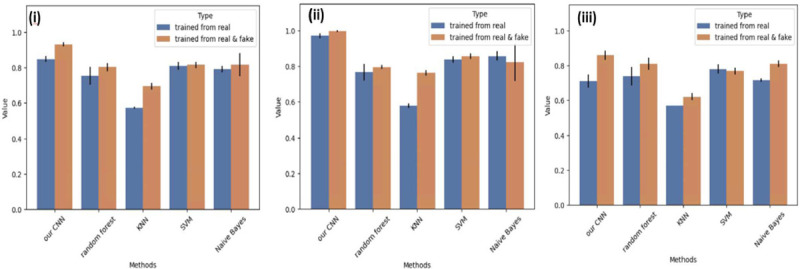
Classification results of our CNN classifier and other state-of-art machine learning algorithms. (A) The classification results of (i) total accuracy of seen domain test and unseen domain tests (ii) accuracy of seen domain results (iii) unseen domain results for each classification method.

To evaluate the performance of our proposed model, we conducted a comparative analysis against other state-of-the-art methods, including Random Forest, KNN, SVM and Naive Bayes. Each method was trained using a small dataset of seen domain real samples as well as a mixed dataset comprising both real and synthetic samples. In order to assess the generalization ability of each method, both seen real data and unseen real data were used for testing. The experiments were conducted with different combinations of training and testing data, and each combination was repeated for 10 cycles. The classification results obtained from the GAN model were compared with those of the other methods, and the results are displayed in Fig. 9 and summarized in Table II.

The results demonstrate that our CNN classifier achieves the highest classification accuracy in both tests that used real data and mixed data respectively for training. Furthermore, it is observed that the accuracy of the seen domain data tests consistently surpasses that of the unseen domain tests, except for the random forest method trained with both real and synthetic data. This observation reinforces the challenge of building a generalized model when working with limited training datasets. Importantly, nearly all of the methods exhibit improved accuracy when synthetic data generated by the GAN model is incorporated to the training. This finding underscores the GAN model’s ability to generate high-quality synthetic data and the rationale behind augmenting the dataset with synthetic samples with respect to sample size and diversity. Overall, these results validate the superior performance of our proposed CNN classifier and emphasize the potential benefits of leveraging synthetic data to enhance classification accuracy, particularly when working with limited training datasets.

**Figure 10.**
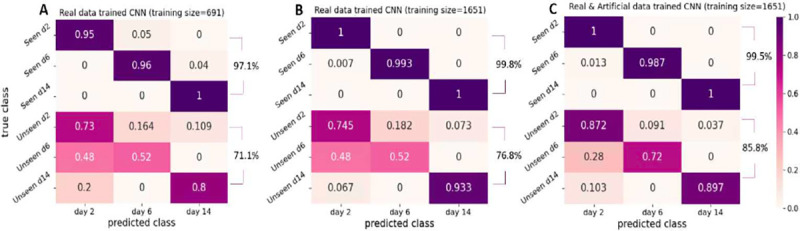
Summary of prediction results of our GAN based classifier

**Figure 11.**
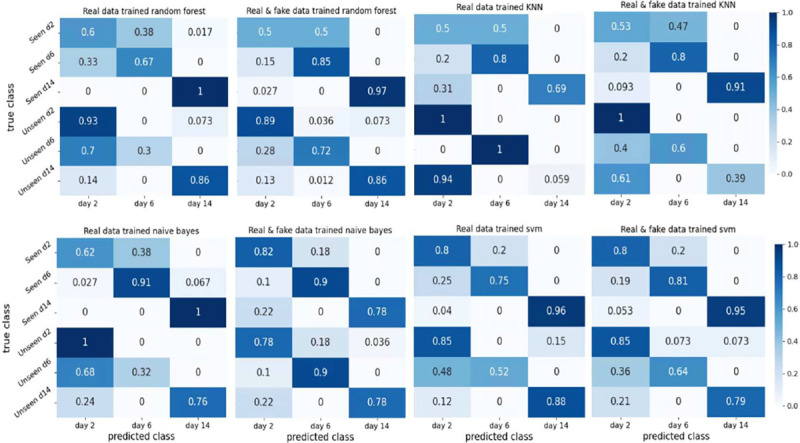
Summary of prediction results of other classifiers

To assess the classification accuracy of each cell class, we generated heatmaps that visualize the performance under different combinations of training and testing data. The results are presented in Fig. 10 and Fig. 11. In Fig. 10, we present the classification results of our proposed CNN classifier trained on three different datasets: a small training dataset that consists of real samples (Fig. 10A), a larger dataset that consists of real samples (Fig. 10B), and a mixed dataset containing both real and synthetic samples (Fig. 10C). Comparing Fig. 10A and Fig. 10B, it is evident that utilizing a larger real dataset leads to improved classification accuracy in both seen domain and unseen domain tests. This improvement can be attributed to increased number of samples, allowing for a more comprehensive coverage of the distinctive characteristics exhibited by the different cell classes. Comparison between Fig. 10B and Fig. 10C also reveals improved performance of the unseen data test. This finding verifies that incorporation of synthetic data generated by the GAN model results in a more robust dataset, which can better handle potential perturbations in different domain data and extend the classifier’s potential to generalize beyond the original seen domain. However, when comparing Fig. 10B to Fig. 10C, we observe that there is no significant improvement in the accuracy of seen data tests. This finding suggests that the seen data tests already achieve a high level of accuracy, leaving little room for further improvement.

The heatmaps depicted in Fig. 10 and Fig. 11 demonstrate that the highest accuracy is achieved in distinguishing hiPSC-CMs images cultured for 14 days, while the classification of day 2 and day 6 cell images often led to misclassification between these two classes. This observation aligns with our earlier PCA results illustrated in Fig. 9, where the day 2 and day 6 data exhibited overlapping distributions of the main principal components, whereas the day 14 data displayed a distinct distribution pattern.

Overall, these observations demonstrate that increasing the size of the real dataset and augmenting it with synthetic data from the GAN model contribute to improved classification accuracy, particularly in scenarios involving unseen domain data. By leveraging a combination of real and synthetic data, our proposed CNN classifier exhibits enhanced robustness and adaptability, making it capable of accurately classifying cells with respect to culture stage or maturation level even in challenging situations.

## V. CONCLUSION

Here, we present the development of a Generative Adversarial Network (GAN) model to generate high quality synthetic data that replicates intact and maturity-enhanced hiPSC-CMs. Synthetic cardiac cell images were generated using the GAN model and combined with an authentic experimental dataset to train a Convolutional Neural Network (CNN) model. The performance of the model was evaluated using an unseen domain dataset, and the results demonstrate that incorporating synthetic data significantly improves accuracy of classifying cells into distinct temporal stages in the maturation process. Principal Component Analysis (PCA) confirmed the GAN model’s ability to extract important features and introduce novel characteristics that may have been hidden in the original data. The proposed model outperformed four conventional machine learning algorithms such as random forest, KNN, SVM and Naive Bayes, and the improvement of the model’s generalization ability by incorporating synthetic data is verified in each of these state-of-art models. The analysis emphasizes the difficulties in developing a classifier that can classify samples with limited training data. It also demonstrates the importance of integrating synthetic data in situations where there are limited samples and thus, effectively addresses the challenges imposed by data availability.

## Figures and Tables

**Table I.** Ablation Test on models that share similar structures

		Test accuracy (%)	
Model	Structure	Seen domain	Unseen domain
Proposed CNN Classifier	Input: (3,96,96)	99.5	85.8
	CNN 1: (32,48,48)		
	CNN 2: (16,24,24)		
	CNN 3: (4, 12, 12)		
	FC: (576,64,3)		
Fully Connected Layers	Input: (3,96,96)	77.4	74.2
	FC: (27648,4096,1024,256,64,3)		
Smaller CNN Classifier	Input: (3,96,96)	96.8	65.4
	CNN 1: (16,32,32)		
	CNN 2: (4,8,8)		
	FC: (256,32,3)		
Larger CNN Classifier (same as discriminator)	Input: (3, 128, 128)	100	77.5
	CNN 1: (32,48,48)		
	CNN 2: (16,24,24)		
	CNN 3: (4, 12, 12)		
	FC: (576, 64, 3)		

**Table II.** Summary of classification results of different combinations of the training and testing data

Data type	Test type	Our CNN	Random Forest	KNN	SVM	Naive Bayes
Trained from real	Seen data test	97.1	76.7	57.9	83.8	85.7
	Unseen data test	71.1	73.7	56.8	77.9	71.6
	Total accuracy	84.7	75.2	57.4	81.0	78.9
Trained from real & fake	Seen data test	99.5	79.5	76.2	85.7	82.1
	Unseen data test	85.8	81	62.1	76.4	80.8
	Total accuracy	92.9	80.2	69.5	81.5	81.6
